# Grau de implantação da segurança do paciente na atenção primária à saúde de três capitais brasileiras

**DOI:** 10.1590/0102-311XPT014824

**Published:** 2025-06-27

**Authors:** Taise Rocha Macedo, Maria Cristina Marino Calvo

**Affiliations:** 1 Secretaria de Estado da Saúde de Santa Catarina, Florianópolis, Brasil.; 2 Universidade Federal de Santa Catarina, Florianópolis, Brasil.

**Keywords:** Segurança do Paciente, Atenção Primária à Saúde, Avaliação em Saúde, Patient Safety, Primary Health Care, Health Evaluation, Seguridad del Paciente, Atención Primaria de Salud, Evaluación en Salud

## Abstract

A segurança do paciente busca a redução de riscos e danos relacionados aos cuidados em saúde e precisa ser implantada no âmbito da atenção primária para qualificar a segurança do cuidado prestado. Objetivou-se determinar o grau de implantação da segurança do paciente na atenção primária das capitais do Sul do Brasil. Trata-se de uma pesquisa avaliativa - do tipo “análise de implantação” por meio de casos múltiplos - desenvolvido nas três capitais sulistas. A segurança do paciente está parcialmente implantada nas capitais do Sul do Brasil (46,6%, 62,5% e 53,3%), não sendo identificado um componente com implantação satisfatória nos casos avaliados. O componente “processos assistenciais” obteve a melhor pontuação (62,5%, 69,2% e 57,5%), e o componente “educação permanente” foi o que obteve os menores percentuais de implantação (33,7%, 56,7% e 27,5%). Os componentes “recursos”, “processos assistenciais”, “cultura de segurança” e “educação permanente” precisam ser aprimorados e fortalecidos para que a segurança do paciente seja implantada e promova cuidados de saúde mais seguros na atenção primária à saúde.

## Abstract (en)

Patient safety is concerned with reducing risks and harm related to healthcare and must be implemented within primary care to improve the quality of care provided. This study aimed to determine the degree of implementation of patient safety in primary care in the capitals of Southern Brazil. This is an evaluative study employing an implementation analysis via multiple case studies, conducted in the capitals of each state in Southern Brazil. Patient safety was found to be partially implemented in these capitals (46.6%, 62.5% and 53.3%), with no component achieving a satisfactory level of implementation in the evaluated cases. The care processes component obtained the highest scores (62.5%, 69.2% and 57.5%), whereas the continuing education component recorded the lowest percentages of implementation (33.7%, 56.7% and 27.5%). The components of resources, care processes, safety culture, and ongoing education need to be strengthened to fully implement patient safety and promote safer healthcare within primary care.

## Abstract (es)

La seguridad del paciente busca reducir riesgos y daños relacionados con la atención médica y debe implementarse dentro del alcance de la atención primaria para tener una calificada seguridad de la atención. El objetivo de este estudio fue determinar el grado de implementación de la seguridad del paciente en atención primaria en las capitales del Sur de Brasil. Se trata de una investigación evaluativa, del tipo de análisis de implementación, realizada con múltiples casos en las capitales de cada estado de la Región Sur de Brasil. La seguridad del paciente se implementa parcialmente en las capitales del Sur de Brasil (46,6%, 62,5% y 53,3%), y no se identificó ningún componente con implementación satisfactoria en los casos evaluados. El componente de los procesos de atención obtuvo el mejor puntaje (62,5%, 69,2% y 57,5%) y el componente de educación continua presentó los porcentajes más bajos de implementación (33,7%, 56,7% y 27,5%). Los componentes de los recursos, los procesos de atención, la cultura de seguridad y la educación continua deben mejorarse y fortalecerse para que haya implementación de la seguridad del paciente y promoción de una atención médica más segura en la atención primaria de salud.

## Introdução

A segurança do paciente preocupa-se em reduzir riscos e danos evitáveis relacionados aos cuidados em saúde ^1^. Na atenção primária à saúde (APS), a temática passou a ser discutida com maior ênfase a partir de 2012, quando a Organização Mundial da Saúde (OMS) instituiu o grupo *Safer Primary Care Expert Working Group* (Grupo de Trabalho para Cuidados Primários Mais Seguros), com a perspectiva de explorar os riscos, a magnitude e a origem dos erros e danos produzidos na APS ^2^.

No Brasil, os destaques envolvendo a temática foram a publicação da *Portaria nº 529* ^3^, de 1º de abril de 2013, que instituiu o Programa Nacional de Segurança do Paciente (PNSP), e a *Resolução da Diretoria Colegiada nº 36/2013* ^4^, que estabeleceu as ações necessárias para a segurança do paciente nos serviços de saúde, fortalecendo as discussões sobre esta pauta e tornando-a uma demanda prioritária a ser explorada pelos estabelecimentos de saúde brasileiros.

Apesar da importância já atribuída a esta matéria, na APS brasileira o tema só foi incorporado junto à Política Nacional da Atenção Básica (PNAB) em 2017, quando a norma expôs a necessidade de implementar ações para garantir a segurança do paciente e reduzir riscos e eventos adversos na APS, sendo essas ações uma atribuição de todos os profissionais e um assunto transversal para a organização do processo de trabalho e de outras políticas de saúde ^5^.

Os índices de eventos adversos comprovam a relevância da temática. Um estudo brasileiro aponta que a taxa de eventos adversos produzidos na APS é de 0,9%, sendo as falhas na comunicação, no cuidado e na gestão os fatores contribuintes com os incidentes de segurança ^6^. Apesar de parecer um valor irrisório, se considerarmos que entre os meses de janeiro e agosto de 2023 foram realizadas - em média no Brasil - 5,6 milhões de consultas médicas mensais na APS ^7^, sugere-se que cerca de 51 mil pessoas foram vítimas de um evento adverso em cada mês. É um número expressivo mesmo sem considerar as possibilidades de eventos adversos nos atendimentos prestados pelos outros profissionais de saúde nesse ambiente de cuidado.

Destaca-se que as taxas de eventos adversos divergem de acordo com: o país investigado, os setores, a natureza e os níveis de atenção do serviço de saúde ^8^ - assim como a proporção dos danos que podem ser evitados ^9^. Nos países de baixa renda, esses índices chegam a 60% ^9^, demonstrando os esforços que devem ser direcionados à temática. No entanto, as pesquisas seguem direcionadas ao ambiente hospitalar com pouca investigação na APS ^10^. 

Considerando a APS como a porta preferencial de acesso aos serviços de saúde que executa o maior volume de cuidados executados na atenção à saúde, evidencia-se a necessidade de dedicação de esforços para o fortalecimento da temática na APS ^10^. 

Evidências apontam que os componentes estruturais, processuais, de treinamento e de cultura precisam sem monitorados e fortalecidos com a participação da gestão, dos profissionais de saúde e dos próprios pacientes, familiares e cuidadores, a fim de fortalecer as ações bem-sucedidas, e planejar e desenvolver outras que conduzam a APS para um ambiente seguro ^10^ ^,^ ^11^ ^,^ ^12^.

Os estudos avaliativos surgem como uma oportunidade de monitorar e diagnosticar o cenário de implantação do objeto explorado. No caso da segurança do paciente na APS, pode-se viabilizar reflexões e ajustes das estruturas e processos existentes que qualifiquem e garantam a segurança do cuidado prestado. Assim, dada a escassez de tais estudos, o objetivo deste artigo é apresentar uma avaliação do grau de implantação da segurança do paciente na APS de três capitais da Região Sul do Brasil.

## Método

Trata-se de uma pesquisa avaliativa do tipo “análise de implantação” com coleta de dados realizada de novembro a dezembro de 2022, com abordagem quantitativa, utilizando amostra intencional e não probabilística. Desenvolveu-se por meio de um estudo de casos múltiplos a partir da seguinte questão de pesquisa: como está implantada a segurança do paciente na APS das capitais do Sul do Brasil?

Os casos selecionados foram as capitais de cada estado da Região Sul do Brasil e as unidades de análise foram as equipes de saúde da família avaliadas como “ótima” ou “muito boa” na certificação do 3º ciclo do Programa de Melhoria do Acesso e da Qualidade da Atenção Básica (PMAQ-AB), correspondendo a 70 equipes no caso 1, 36 equipes no caso 2 e 36 equipes no caso 3 ^13^. Esse critério foi adotado pelo fato de as classificações “ótima” e “muito boa” indicarem o alcance de padrões estratégicos de acessibilidade, gestão, processo de trabalho e resultados alcançados pelas equipes, sugerindo maior possibilidade de executarem os cuidados pautados na segurança do paciente. 

Os informantes foram os enfermeiros vinculados às equipes de saúde incluídas no estudo, selecionados por serem profissionais que participam ativamente nos processos de qualificação e segurança dos cuidados prestados na APS no nível gerencial e assistencial ^14^. Foram convidados a participar do estudo 157 enfermeiros, dos quais 36 aceitaram participar - assinando o Termo de Consentimento Livre e Esclarecido e respondendo ao instrumento de coleta de dados. Participaram 13 enfermeiros do caso 1, 13 enfermeiros do caso 2 e 10 enfermeiros do caso 3.

Os dados foram obtidos por meio da aplicação de um formulário, construído em consenso com os especialistas que participaram do estudo de avaliabilidade ^15^, que validou os modelos teóricos, lógicos e a matriz de análise e julgamento da segurança do paciente na APS. A modelização revelou os fatores contextuais externos e internos que atuam sobre a segurança do paciente na APS, evidenciando também os componentes imprescindíveis para a sua implantação e que passaram a fazer parte do instrumento da coleta de dados ^15^. 

O formulário foi disponibilizado em uma plataforma online interativa (GoogleForms; https://docs.google.com/forms/u/0/), com perguntas agrupadas pelos componentes que estruturam a segurança do paciente na APS, conforme apresentado na matriz de análise e julgamento utilizada neste estudo: Recursos (13 itens/oito indicadores); Cultura de Segurança (18 itens/cinco indicadores); Processos Assistenciais (seis itens/quatro indicadores); e Educação Permanente (quatro itens/quatro indicadores) (Quadro 1).

**Quadro 1:** Matriz de análise e julgamento da segurança do paciente na atenção primária à saúde das capitais da Região Sul do Brasil, 2023.

COMPONENTE RECURSOS	PEC = 12
Critério/Indicador - fonte de evidência e medida no instrumento	Parâmetros
Estrutura física da UBS de acordo com as normas vigentes	PEI =2
Item 1.2 A UBS possui em sua estrutura física os ambientes: 1.2.1 Área de recepção e/ou espera; 1.2.2 Consultório sem banheiro; 1.2.3 Consultório com banheiro; 1.2.4 Sala de procedimentos; 1.2.5 Sala de curativo; 1.2.6 Depósito de material e limpeza; 1.2.7 Dois sanitários	7 respostas “sim” = 2; 6 ou 5 respostas “sim” = 1; < 5 respostas “sim” = 0
Equipe multiprofissional composta e com formação adequada para atender as necessidades populacionais	PEI = 2
Item 1.3 A ESF é composta por no mínimo um médico, um enfermeiro, um técnico ou auxiliar de enfermagem e um agente comunitário de saúde? Item 1.4 A composição da equipe é adequada para atender as demandas populacionais com segurança? Item 1.5 Os profissionais de nível superior possuem especialização em medicina de família, saúde da família ou saúde coletiva? 1.5.1 Médico; 1.5.2 Enfermeiro; 1.5.3 Cirurgião dentista	1.3 sim = 0,5; 1.4 sim = 0,75; 1.5 = 0,25 cada
Materiais, equipamentos e insumos à disposição das equipes e dos usuários	PEI = 2
Item 1.6 A UBS possui materiais o suficiente para executar com segurança as ações de promoção, prevenção, proteção, diagnóstico, tratamento, reabilitação, redução de danos, cuidados paliativos e vigilância em saúde? Item 1.7 A UBS possui equipamentos o suficiente para executar com segurança as ações de promoção, prevenção, proteção, diagnóstico, tratamento, reabilitação, redução de danos, cuidados paliativos e vigilância em saúde? Item 1.8 A UBS possui insumos o suficiente para executar com segurança as ações de promoção, prevenção, proteção, diagnóstico, tratamento, reabilitação, redução de danos, cuidados paliativos e vigilância em saúde?	3 respostas “sim” = 2 2 respostas “sim” = 1 1 resposta “sim” = 0
Registro eletrônico em saúde com ferramentas de suporte cognitivo e de apoio a decisão clínica	PEI = 1
Item 1.9 A UBS utiliza registro eletrônico? Item 1.10 O sistema utilizado agrega ferramenta tecnológica que fornece alertas em prontuário que apoiam o processo cognitivo e as tomadas de decisões clínicas?	2 respostas “sim”= 1 1 resposta “sim”= 0,5
Sistemas internos de notificação de incidentes local (descentralizado) e/ou centralizado implantado(s)	PEI = 1
Item 1.11 O município possui algum sistema de notificação de incidentes de segurança?: 1.11.1 Caso a resposta anterior seja sim, o acesso está liberado para todos os membros da equipe para efetuar notificação?	2 respostas “sim” = 1 1 resposta “sim” = 0,5
Núcleo de Segurança do Paciente implantado e plano de segurança do paciente instituído	PEI = 2
Item 1.12 O município possui Núcleo de Segurança do Paciente implantado? Item 1.13 O município possui plano de segurança do paciente instituído?	2 respostas “sim” = 2 1 resposta “sim” = 1
Protocolos de segurança do paciente instituído	PEI = 1
Item 1.14 O município possui protocolos de segurança voltados para: 1.14.1 Identificação do paciente?; 1.14.2 Higienização das mãos; 1.14.3 Prevenção de quedas?; 1.14.4 Prevenção de lesão por pressão?; 1.14.5 Segurança dos medicamentos?; 1.14.6 Comunicação efetiva?; 1.14.7 Cirurgia segura?	7 respostas “sim” = 1 6 respostas “sim” = 0,5 < 6 respostas “sim” = 0
Diretrizes clínicas /protocolos assistenciais e plano de monitoramento e avaliação das tecnologias	PEI = 1
Item 1.15 O município possui diretrizes clínicas e/ou protocolos assistenciais implantados?: 1.15.1 As diretrizes clínicas e/ou protocolos assistenciais estão implantados para todas as categorias profissionais? Item 1.16 O município possui plano teórico/prático de monitoramento e avaliação das tecnológicas utilizadas na APS implantado?	1.15 sim = 0,25; 1.15.1 sim = 0,25; 1.16 Sim = 0,5
GII = ((ΣPOI/ΣPEI) x 100)	
COMPONENTE CULTURA DE SEGURANÇA	PEC = 10
Critério/Indicador - fonte de evidência e medida no instrumento	Parâmetros
Avaliação sistemática da cultura de segurança institucional	PEI = 2
Item 2.1 Você já participou de alguma avaliação da cultura de segurança no período em que atua neste município?	Sim = 2; Não = 0
Redução de gradientes de hierarquia	PEI = 2
Item 2.2 Os profissionais têm liberdade para se expressar ao observarem algo que pode afetar negativamente o cuidado ao paciente? Item 2.3 Os profissionais sentem-se à vontade para questionar as decisões ou ações de seus superiores? Item 2.4 Os profissionais sentem-se à vontade para perguntar, quando algo parece não estar certo?	Itens 2.2.e 2.3: Sim = 0,75; Em parte = 0,25; Não = 0; Item 2.4: Sim = 0,5; Em parte = 0,25; Não = 0
Cultura justa	PEI =2
Item 2.5 Os profissionais consideram que seus erros, enganos ou falhas podem ser usados contra eles? Item 2.6 Quando um evento é relatado, o foco recai sobre a pessoa e não sobre o problema?	Cada item: Não = 1; Em parte = 0,5; Sim = 0
Liderar e apoio a equipe	PEI =2
Item 2.7 Os processos e sistemas utilizados na APS são adequados para prevenir a ocorrência de erros? Item 2.8 As sugestões de melhoria da segurança do paciente emitida pelos profissionais são discutidas/acatadas? Item 2.9 Existe na unidade de saúde cartaz, *folder*, *poster* sensibilizando profissionais e pacientes para a temática da segurança do paciente?	Itens 2.7 e 2.8: Sim = 0,75; Em parte = 0,25; Não = 0 Item 2.9: Sim = 0,5; Em parte ou Não = 0
Identificação, análise, avaliação, monitoramento e comunicação de riscos de segurança	PEI = 2
Item 2.10 São realizadas auditorias internas para identificar situações de risco ou oportunidades de melhorias relacionada a segurança do paciente? Item 2.11 São sempre utilizadas as informações das queixas e reclamações (ouvidoria) para identificação de riscos de segurança? Item 2.12 São desenvolvidas observações diretas com o objetivo de identificar riscos de segurança na APS? Item 2.13 Existe mapeamento de riscos de segurança nas UBS? Item 2.14 Quando um incidente de segurança acontece, ele é discutido com a equipe de saúde envolvida? Item 2.15 Os incidentes são notificados e implicam em revisão dos processos do serviço e a implantação de melhorias? Item 2.16 São utilizadas ferramentas de análise e avaliação do risco institucional? Item 2.17 Todas as mudanças implementadas em prol da segurança do paciente são comunicadas à equipe de saúde? Item 2.18 O desempenho da segurança do paciente na APS é monitorado por meio de indicadores?	> 7 respostas “sim” = 2; 5 ou 6 respostas “sim” = 1,0; < 5 respostas “sim” = 0
GII = ((ΣPOI/ ΣPEI) x 100)	
COMPONENTE PROCESSOS ASSISTENCIAIS	PEC = 8
Critério/Indicador - fonte de evidência e medida no instrumento	Parâmetros
Adesão às normativas clínicas e de segurança	PEI = 2
Item 3.1 Todos os profissionais da equipe de saúde conhecem e utilizam os protocolos de segurança do paciente? Item 3.2 Todos os profissionais da equipe de saúde conhecem e utilizam os protocolos institucionais?	2 respostas “sim” = 2; 1 resposta “sim” = 1; Nenhuma resposta “sim” = 0
Paciente, família e/ou cuidador participando ativamente do cuidado	PEI = 2
Item 3.3 O cuidado desenvolvido pelos profissionais sempre é centrado no paciente/familiar/cuidador?	Sim = 2; Não = 0
Erros e/ou eventos adversos notificados	PEI =2
Item 3.4 Sobre a notificação de incidentes de segurança pela equipe de saúde da APS, assinale a alternativa correspondente (apenas 1 opção): 3.4.1 Todos os incidentes de segurança são notificados; 3.4.2 Todos os incidentes de segurança que atingiram os pacientes são notificados; 3.4.3 Nem todos são notificados	Sim para o item 3.4.1 = 2; Sim para o item 3.4.2 = 1; Sim para o item 3.4.3 = 0
Transição de cuidado	PEI =2
Item 3.5 Todos os profissionais se preocupam em garantir uma comunicação adequada com o paciente, familiar/cuidador e com os demais profissionais da equipe ou da rede de atenção à saúde? Item 3.6 A UBS utiliza adequadamente o sistema de referência, reportando os casos de maneira completa aos diferentes pontos de atenção à saúde?	2 respostas “sim” = 2; Demais = 0
GII = ((ΣPOI/ ΣPEI) x 100)	
COMPONENTE EDUCAÇÃO PERMANENTE	PEC = 8
Critério/Indicador - fonte de evidência e medida no instrumento	Parâmetros
Profissionais capacitados para a segurança do paciente	PEI = 2
Item 4.1 Você participou de alguma capacitação em segurança do paciente nos últimos 12 meses?	Sim = 2; Não = 0
Profissionais capacitados para o cuidado seguro	PEI = 2
Item 4.2 Os profissionais participam de um processo de qualificação profissional contínuo por meio da educação continuada e/ou permanente?	Sim = 2; Não = 0
Pacientes capacitados para a segurança do paciente	PEI = 2
Item 4.3 São desenvolvidas ações ou capacitações para os pacientes, conscientizando-os sobre a temática da segurança?	Sim = 2; Não = 0
Ações direcionadas à saúde do trabalhador	PEI = 2
Item 4.4 Sobre as ações direcionadas à saúde dos trabalhadores da(s) equipe(s) de saúde: 4.4.1 São desenvolvidas ações de promoção à saúde dos trabalhadores da equipe?; 4.4.2 São desenvolvidas ações direcionadas à proteção da saúde dos trabalhadores da equipe?; 4.4.3 São desenvolvidas ações direcionadas à recuperação e reabilitação à saúde dos trabalhadores da equipe?	3 respostas “sim” = 2; 2 respostas “sim” = 1; < 2 respostas “sim” = 0
GIC = ((ΣPOC/ ΣPEC) x 100)	
GISP na APS = ((ΣPOI Recursos*2) + ΣPOI Cultura de Segurança + ΣPOI Processos Assistenciais + ΣPOI Educação Permanente/ΣPEISP) x 100)	

APS: atenção primária à saúde; GIC: grau de implantação do componente; GII: grau de implantação do indicador da segurança do paciente; GISP: grau de implantação da segurança do paciente; PEC: pontuação esperada no componente; PEI: pontuação esperada no indicador; PEISP: pontuação esperada para implantação da segurança do paciente; POC: pontos observados no componente; POI: pontuação obtida no indicador; UBS: unidade básica de saúde.

Os participantes responderam a 44 itens vinculados a 22 indicadores da segurança do paciente. No entanto, em razão do desconhecimento dos participantes aos três itens que formam o indicador “Orçamento para as ações de segurança do paciente” vinculado ao componente Recursos, ele foi retirado da análise e julgamento.

A pontuação obtida no indicador (POI) resulta do somatório dos pontos obtidos em cada grupo de itens do formulário aplicado e corresponde à avaliação do indicador. Ao final de cada grupo de indicadores apresenta-se a pontuação máxima esperada e a pontuação obtida no componente, que corresponde ao somatório da pontuação obtida nos indicadores do grupo.

O grau de implantação do indicador da segurança do paciente (GII) resulta no percentual do somatório dos POI em relação à pontuação esperada no indicador (PEI):

GII=(ΣPOI/ ΣPEI)×100

O mesmo cálculo é aplicado para o grau de implantação do componente (GIC), que é a proporção do somatório dos pontos observados no componente (POC) em relação à pontuação esperada no componente (PEC)

GIC=(Σ POC/ ΣPEC)×100

Para a análise final do grau de implantação da segurança do paciente (GISP) na APS, atribuiu-se peso dois (2) para o componente Recursos e peso um (1) para os demais componentes. Essa ponderação decorre do pressuposto de que os recursos são essenciais para que se tenha maior possibilidade de implantação dos demais componentes da segurança do paciente.

O cálculo utilizado para definir o GISP corresponde à soma das pontuações observadas nos indicadores recurso (POI Recursos), considerando seu peso diferenciado para esta avaliação, a cultura de segurança (POI Cultura de Segurança), os processos assistenciais (POI Processos Assistenciais) e a educação permanente (POI Educação Permanente) em relação à soma da pontuação esperada para implantação da segurança do paciente (PEISP)

$$GISP=[\left(\Sigma POI\;Recursos*2\right)+\Sigma POI\;Cultura\;de\;Segurança+\Sigma POI\;Processos\;Assistenciais+\Sigma POI\;Educação\;Permanente/\Sigma PEISP)\times 100]$$

Os indicadores, os componentes e os casos foram estratificados em três níveis de julgamento, quais sejam: < 50% implantação incipiente, valores ≥ 50% e < 75% implantação parcial e valores ≥ 75% implantação satisfatória.

O estudo foi aprovado pelo Comitê de Ética em Pesquisa com Seres Humanos da Universidade Federal Santa Catarina (parecer nº 5.666.994/2022).

## Resultados

Os resultados encontrados foram transpostos para a Tabela 1, que apresenta e detalha o conjunto de indicadores utilizados na avaliação da segurança do paciente na APS e o grau de implantação em cada um dos indicadores e componentes avaliados em cada um dos casos.

Para o componente Recursos, os indicadores “Estrutura física da unidade de saúde de acordo com as normas vigentes” e “Registro eletrônico em saúde com ferramentas de suporte cognitivo e de apoio à decisão clínica” tiveram maiores pontuações. Já os indicadores “Núcleo de Segurança do Paciente implantado e plano de segurança do paciente instituído” e “Protocolos de segurança do paciente instituído” obtiveram as mais baixas pontuações. Dentre os casos analisados, o caso 1 obteve a pontuação mais baixa no componente Recursos (Tabela 1).

O componente Cultura de Segurança apresentou as maiores pontuações no indicador “Reduzir gradientes de hierarquia”, enquanto as menores pontuações foram localizadas nos indicadores “Avaliação sistemática da cultura de segurança institucional” e “Identificar, analisar, avaliar, monitorar e comunicar os riscos de segurança”. O caso 1 teve o pior desempenho deste componente (Tabela 1).

Em Processos Assistenciais, o indicador “Paciente, família e/ou cuidador participando ativamente do cuidado” obteve os valores mais altos dentre todos os indicadores analisados. Já os indicadores “Adesão as normativas clínicas e de segurança” e “Erros e/ou eventos adversos sendo notificados” foram os que receberam as piores avaliações (Tabela 1). 

No componente Educação Permanente, o indicador com maior percentual de implantação foi “Profissionais capacitados para o cuidado seguro” no caso 2. Já os indicadores “Profissionais capacitados para a segurança do paciente” e “Pacientes capacitados para a segurança do paciente” foram os que obtiveram os menores percentuais de implantação (Tabela 1).

A Tabela 1 também apresenta o grau de implantação de cada componente em cada uma das capitais do Sul do Brasil e a análise final do grau de implantação da segurança do paciente de cada capital. A melhor implantação da segurança do paciente na APS, de acordo com a avaliação e desempenho observado, foi identificado no caso 2 (62,5%).

**Tabela 1:** Grau de implantação (%) dos indicadores e componentes da segurança do paciente na atenção primária à saúde nas capitais da Região Sul do Brasil, 2023.

Componente/Indicador	Caso 1 (%)	Caso 2 (%)	Caso 3 (%)
Recursos			
Estrutura física da UBS de acordo com as normas vigentes	76,9	73,1	85,0
Equipe multiprofissional composta e com formação adequada para atender às necessidades populacionais	67,3	71,2	67,5
Materiais, equipamentos e insumos à disposição das equipes e dos usuários	46,2	61,5	45,0
Registro Eletrônico em Saúde com ferramentas de suporte cognitivo e de apoio à decisão clínica	65,4	92,3	80,0
Sistemas internos de notificação de incidentes local (descentralizado) e/ou centralizado implantado (s)	50,0	50,0	50,0
Núcleo de Segurança do Paciente implantado e plano de segurança do paciente instituído	19,2	50,0	45,0
Protocolos de segurança do paciente instituído	19,2	50,0	60,0
Diretrizes clínicas/protocolos assistenciais e plano de monitoramento e avaliação das tecnologias utilizadas no âmbito da APS	44,2	63,5	70,0
GIC Recursos (peso 2)	49,8	63,9	62,1
Cultura de Segurança			
Avaliação sistemática da cultura de segurança institucional	7,7	38,5	30,0
Redução de gradientes de hierarquia	69,2	84,6	71,3
Promoção de uma cultura justa	61,5	67,3	50,0
Liderança e apoio à equipe	32,7	53,8	41,3
Identificação, análise, avaliação, monitoramento e comunicação de riscos de segurança	11,5	46,2	55,0
GIC Cultura de Segurança	36,5	58,1	49,5
Processos Assistenciais			
Adesão às normativas clínicas e de segurança	46,2	61,5	45,0
Paciente, família e/ou cuidador participando ativamente do cuidado	100,0	92,3	90,0
Erros e/ou eventos adversos sendo notificados	50,0	53,8	45,0
Qualificar o processo de transição de cuidado	53,8	69,2	50,0
GIC Processos Assistenciais	62,5	69,2	57,5
Educação Permanente			
Profissionais capacitados para a segurança do paciente	7,7	61,5	0,0
Profissionais capacitados para o cuidado seguro	69,2	84,6	40,0
Pacientes capacitados para a segurança do paciente	15,4	30,8	10,0
Desenvolvimento de ações direcionadas à saúde do trabalhador	42,3	50,0	60,0
GIC Educação Permanente	33,7	56,7	27,5
GISP na APS	46,6	62,5	53,3

APS: atenção primária à saúde; GIC: grau de implantação do componente; GISP: grau de implantação da segurança do paciente; UBS: unidade básica de saúde.

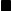
≤ 50% incipiente

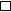
50% a 75% parcial

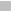
≥ 75% satisfatório

## Discussão

Este estudo aponta que a segurança do paciente na APS está parcialmente implantada nas capitais do Sul do Brasil. O componente Recursos, composto por indicadores que avaliam requisitos mínimos para a oferta de cuidados seguros na APS - como estrutura física, equipe, materiais, equipamentos, insumos, tecnologia e normativas que condicionam inclusive a execução satisfatória dos demais componentes que estruturam a segurança do paciente na APS -, está implantado de modo incipiente e parcial nos casos avaliados.

Esses itens devem receber muita atenção, uma vez que a literatura destaca que a infraestrutura, a composição das equipes vinculadas às unidades de saúde, os recursos e os insumos disponibilizados aos profissionais e pacientes - aliados aos aspectos tecnológicos - são barreiras para a ocorrência de incidentes de segurança e, quando não disponibilizados adequadamente, contribuem para a ocorrência de eventos adversos ^6^ ^,^ ^8^ ^,^ ^16^ ^,^ ^17^. 

A baixa implantação de Núcleos de Segurança do Paciente (NSP) - identificada neste estudo - suscita um cenário de insegurança na APS e pode estar atrelada a uma fragilidade quanto ao compromisso institucional/gestão diante da segurança do paciente ^18^. No Brasil, o NSP é a estrutura responsável por implementar e gerir as ações de melhoria da qualidade da segurança do paciente por meio do plano de segurança do paciente. Além disso, os NSP colaboram nos processos de implantação dos protocolos de segurança e em outras questões institucionais que venham a ser demandados pelos serviços, como os protocolos assistenciais/as diretrizes clínicas, necessários para a qualificação do cuidado executado ^17^ ^,^ ^19^, sendo uma estrutura imprescindível para o fortalecimento de uma política institucional de segurança.

Os NSP continuam mais presentes nos estabelecimentos hospitalares - sendo pouco presentes na APS. Essa experiência do contexto hospitalar sugere estratégias que podem auxiliar a implantação dos NSP na APS ^20^. Algumas barreiras para a implantação dos NSP já vivenciadas pelos hospitais e apontadas pela literatura incluem: a limitação dos recursos financeiros, o desconhecimento quanto ao processo de implantação desta estrutura e uma cultura de segurança frágil ^18^. 

A cultura de segurança, o fruto dos valores, as crenças e as atitudes individuais e coletivas vinculadas à segurança do paciente em uma organização ^11^ são componentes consolidados na literatura - com instrumentos validados e recomendações para sua investigação sistemática. Expõem áreas frágeis e oportunizam intervenções para o fortalecimento sobre elas, sendo um componente fundamental para a melhoria da segurança do paciente e qualificação dos cuidados em saúde ^8^ ^,^ ^18^. No âmbito da APS, já foi explorada no Brasil ^10^ ^,^ ^21^ e em outros países como Grécia ^11^ e Tunísia ^8^.

Neste estudo, dentre os indicadores avaliados na Cultura de Segurança, os melhores percentuais de implantação foram encontrados em gradientes de hierarquia - sugerindo que, nos casos explorados, a distância psicológica entre o trabalhador e o seu supervisor acontece de modo natural, sem excessos, encorajando os indivíduos e oportunizando compartilhamento de preocupações, conhecimentos e experiências ^22^. Baixos gradientes de hierarquia favorecem o trabalho em equipe e fortalecem a cultura de segurança ^11^. 

Já os indicadores com os piores desempenhos tratam da gestão de risco e da avaliação sistemática da cultura institucional. Na Tunísia ^8^, um resultado semelhante a este também foi encontrado em uma avaliação da cultura de segurança da APS local, quando o indicador que avalia a frequência de eventos notificados recebeu baixas pontuações. A gestão de risco preocupa-se em planejar, monitorar e comunicar os riscos e os eventos adversos, contribuindo para um ciclo de melhorias de segurança, oportunizando planejar intervenções nas ações geradoras de erros, e consequentemente produzir uma gestão de risco eficaz, que atua quando as falhas e os danos ainda podem ser evitados ^8^ ^,^ ^23^.

A literatura destaca alguns elementos que facilitam o processo de notificação dos eventos adversos, quais sejam: apoio institucional, cultura de segurança institucional, aprimoramento do sistema de notificação e incentivo ao relato voluntário e confidencial. Entretanto, alguns elementos dificultadores também foram mencionados e se trata da falta de recursos materiais/humanos; do medo/vergonha; da postura institucional punitiva; da falta de estímulo e das lacunas no conhecimento ^23^. 

No componente Processos Assistenciais, explora-se o envolvimento dos profissionais da equipe de saúde com a temática da segurança do paciente no provimento de ações na APS, sendo o componente que apresentou o maior percentual de implantação dentre os avaliados.

O indicador com destaque positivo trata da participação do paciente, família e/ou cuidador no processo de cuidado. Esse resultado atende as recomendações do PNSP e da Organização Mundial da Saúde (OMS) ^24^, que evidenciam que o envolvimento do paciente, família e/ou cuidador otimiza recursos, promove corresponsabilidades no processo de cuidado, gera aprendizados e melhorias que impactam nos resultados de saúde e na redução de eventos adversos, uma vez que complementam a visão dos profissionais de saúde, devendo ser cada vez mais fortalecido e aprimorada na APS ^3^ ^,^ ^24^ ^,^ ^25^.

Apesar da literatura demostrar que os profissionais afirmam incluir o paciente no seu tratamento e na sua própria segurança ^17^, os incidentes em serviços de saúde notificados por cidadãos no Sistema de Notificação de Incidentes da Agência Nacional de Vigilância Sanitária (Anvisa) observou baixa participação dos pacientes no sistema de notificação - sendo que apenas 5,3% das notificações foram realizadas por paciente/familiar/cuidador no contexto da APS ^25^, dado semelhante ao estudo desenvolvido na Inglaterra e na Austrália ^26^.

Os incidentes relatados pelo paciente/familiar/cuidador divergem daqueles relatados pelos profissionais e complementam as fontes de informações, que contribuem para o desenvolvimento de ações que fortalecerão a segurança do paciente. Apesar das baixas notificações parecerem ser um fenômeno generalizado, a participação do paciente no seu cuidado requer envolvê-lo neste processo gerador de conhecimento ^26^. 

Em relação aos indicadores avaliados neste estudo que exploram a adesão a normas clínicas e de segurança, o processo de notificação de erros e eventos adversos e a transição do cuidado nos pontos de atenção precisam ser fortalecidos na APS. A literatura aponta que a implantação e implementação dos protocolos assistenciais e de segurança colaboram para a redução do risco de danos e qualificam a abordagem aos pacientes. O conhecimento e as evidências cientificas compartilhados nas capacitações e nas qualificações de toda a equipe transformam as práticas de trabalho na APS, qualificam a transição dos cuidados dos pacientes nos pontos de atenção à saúde e ajudam a implementar a segurança do paciente nas ações e nos cuidados realizados no dia a dia ^21^. O PNSP aponta como estratégia para sua implementação o processo de capacitação dos profissionais vinculados aos estabelecimentos de saúde - gestores, profissionais ou equipes de saúde -, juntamente com a utilização de protocolos, guias e manuais de segurança do paciente ^4^.

Neste sentido, a educação permanente surge como uma prática fundamental para a construção de uma assistência qualificada e segura, uma vez que vislumbra a interação de atores como gestores, profissionais de saúde e pacientes, com vistas a construção ou reconstrução de conhecimentos ^27^. Apesar das recorrentes recomendações de programas e da literatura ^4^ ^,^ ^12^ ^,^ ^27^ os resultados deste estudo apontam uma implantação parcial do componente, denunciando a falta de preocupação com capacitações vinculadas à temática da segurança do paciente para os profissionais e para os próprios pacientes. 

Dentre as estratégias que podem ser utilizadas para o fortalecimento da Educação Permanente, aponta-se as capacitações e encontros no próprio ambiente de trabalho, transpondo a temática da segurança para as situações do dia a dia; tutoria por especialistas ou profissionais da própria equipe; gravações de casos para discussões e análises; elaboração de portfólios, análise de casos clínicos e simulação clínica ^17^. Intervenções para fortalecimento deste componente são necessárias uma vez que a ausência de capacitações para a temática da segurança interfere no fortalecimento dos demais componentes da segurança do paciente e compromete a atuação dos envolvidos, sejam gestores, profissionais ou paciente na dinâmica e no próprio processo de trabalho da APS ^11^.

Cabe mencionar que a pandemia de COVID-19 impôs limitações para a execução da pesquisa, uma vez que restringiu a execução de estudos presenciais, bem como a interação direta com os participantes. A sobrecarga de trabalho, fadiga física e psicológica impactou na participação dos enfermeiros nesta pesquisa. O estabelecimento de contatos telefônicos e virtuais, bem como a aplicação do formulário de coleta de dados autopreenchível - de maneira remota -, foram estratégias adotadas para amenizar estas barreiras. Também não foram explorados os contextos de implantação dos casos participantes que podem ser motivo de novos estudos.

## Considerações finais

O presente estudo identificou que a segurança do paciente está implantada de forma parcial na APS das capitais da Região Sul do Brasil, sendo necessário o fortalecimento dos componentes - Recursos, Cultura de Segurança, Processos Assistenciais e Educação Permanente - avaliados nesta análise. É essencial que haja um esforço contínuo de todas as partes envolvidas - gestão, profissionais e pacientes - para afirmar a implantação satisfatória destes componentes, garantindo segurança e qualidade nos cuidados executados na APS.

A presença do núcleo de segurança e dos protocolos institucionais, sejam estes clínicos e/ou de segurança, são ações que precisam estar presentes no âmbito da APS para garantir a implantação da segurança do paciente, demandando previsões orçamentárias para este fim.

Outras ações precisam acontecer, como a adesão dos profissionais às normativas clínicas e de segurança e um processo de notificação dos eventos adversos vinculados à APS. Essas atividades precisam ser encorajadas pois só serão efetivadas a partir do reconhecimento da importância que possuem para o fortalecimento da segurança na APS.

A cultura de segurança também necessita ser sistematicamente avaliada, por meio de instrumentos já validados, que subsidiarão intervenções para robustecer a segurança em um dado contexto, uma vez que promove um comprometimento coletivo sobre a pauta em questão. Por isso, estratégias de educação permanente - como os treinamentos realizados dentro da própria rotina das unidades de saúde - despontam como um importante aliado para capacitação e conscientização dos profissionais e do próprio paciente/familiar sobre a temática da segurança e do cuidado seguro.

A segurança do paciente é uma preocupação crescente no contexto da APS, com desafios a serem superados para sua implantação. É essencial que haja um esforço coletivo e contínuo de todas as partes envolvidas - incluindo as instituições de saúde/gestores, os profissionais e os pacientes/familiares/cuidadores - para garantir a segurança e a qualidade dos cuidados de saúde na APS.
